# The reference genome of an endangered Asteraceae, *Deinandra increscens* subsp. *villosa*, endemic to the Central Coast of California

**DOI:** 10.1093/g3journal/jkae117

**Published:** 2024-06-07

**Authors:** Susan L McEvoy, Rachel S Meyer, Kristen E Hasenstab-Lehman, C Matt Guilliams

**Affiliations:** Department of Conservation and Research, Santa Barbara Botanic Garden, Santa Barbara, CA 93105, USA; Department of Ecology and Evolutionary Biology, University of California Santa Cruz, Santa Cruz, CA 95064, USA; Department of Conservation and Research, Santa Barbara Botanic Garden, Santa Barbara, CA 93105, USA; Department of Conservation and Research, Santa Barbara Botanic Garden, Santa Barbara, CA 93105, USA

**Keywords:** *Deinandra increscens* subsp. *villosa*, Madiinae, Asteraceae, Compositae, endemic, endangered, conservation, whole genome duplication, tandem genes

## Abstract

We present a reference genome for the federally endangered Gaviota tarplant, *Deinandra increscens* subsp. *villosa* (Madiinae, Asteraceae), an annual herb endemic to the Central California coast. Generating PacBio HiFi, Oxford Nanopore Technologies, and Dovetail Omni-C data, we assembled a haploid consensus genome of 1.67 Gb as 28.7 K scaffolds with a scaffold N50 of 74.9 Mb. We annotated repeat content in 74.8% of the genome. Long terminal repeats (LTRs) covered 44.0% of the genome with *Copia* families predominant at 22.9% followed by *Gypsy* at 14.2%. Both *Gypsy* and *Copia* elements were common in ancestral peaks of LTRs, and the most abundant element was a *Gypsy* element containing nested *Copia/Angela* sequence similarity, reflecting a complex evolutionary history of repeat activity. Gene annotation produced 33,257 genes and 68,942 transcripts, of which 99% were functionally annotated. BUSCO scores for the annotated proteins were 96.0% complete of which 77.6% was single copy and 18.4% duplicates. Whole genome duplication synonymous mutation rates of Gaviota tarplant and sunflower (*Helianthus annuus*) shared peaks that correspond to the last Asteraceae polyploidization event and subsequent divergence from a common ancestor at ∼27 MYA. Regions of high-density tandem genes were identified, pointing to potentially important loci of environmental adaptation in this species.

## Teaser

McEvoy *et al*. introduce a scaffold-quality reference genome for the endangered Gaviota tarplant. The assembly is 1.67 Gbp with 96% BUSCO completeness, 33 K genes and 75% repeat content. The authors find extensive Copia long terminal repeat sequences and duplicate genes that suggest environmental adaptation strategies. Comparisons with sunflower suggest a shared polyploidization event around 27 million years ago, close to the date of the common ancestor divergence. This work underlines the importance of genomic studies in accurately understanding adaptations and conservation needs.

## Introduction

Genetic analyses can play a critical role in the conservation of rare and endangered species (Theissinger *et al*. 2023). Regulatory agencies, land managers, and conservation biologists are increasingly turning to genetic analyses to inform decisions about the species they manage (Segelbacher *et al*. 2022). Management decisions take on new importance in light of the increasing threats to global biodiversity, such as the climate crisis (Scheffers *et al*. 2016), continued worldwide habitat destruction (González *et al*. 2020), and the spread of invasive species (North *et al*. 2021). Extending beyond traditional population genetic analyses, genome-scale data are being applied within a conservation framework. Genomic data result in more accurate characterization of genetic diversity and enable analyses dependent on genomic coordinates, functional annotations, and examination of features outside of the gene space, such as structural variants and regulatory elements (Fuentes-Pardo and Ruzzante 2017). Reference genomes combined with resequencing across a species’ range are being used, for example, to designate conservation units for the critically endangered *Artocarpus nanchuanensis* (Xia *et al*. 2023), to guide the selection of adaptive traits such as drought resistance in *Fagus sylvatica* (European beech; Pfenninger *et al*. 2021), to understand the adaptive potential in crop wild relatives (Wambugu and Henry 2022), and to aid in the restoration of locally adapted *Castanea dentata* (American chestnut; Sandercock *et al*. 2022). Conversation genomics will gain importance as sequencing technology becomes more efficient and less expensive, computational resources and analytical techniques advance, and existing data and methodological resources grow via concerted efforts such as the Earth BioGenome Project (Lewin *et al*. 2018; Exposito-Alonso *et al*. 2020).

**Fig. 1.**
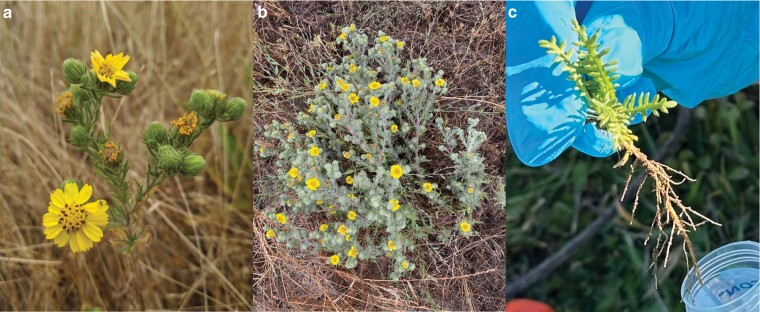
Examples of *D. increscens* subsp. *villosa*, which, as an annual, can vary in form. a) Close-up of the inflorescence on a mature but smaller plant habit. b) An example of the larger plant habit. c) Seedling shoot and roots collected for RNA-seq. Photos courtesy of C. Matt Guilliams.

**Fig. 2.**
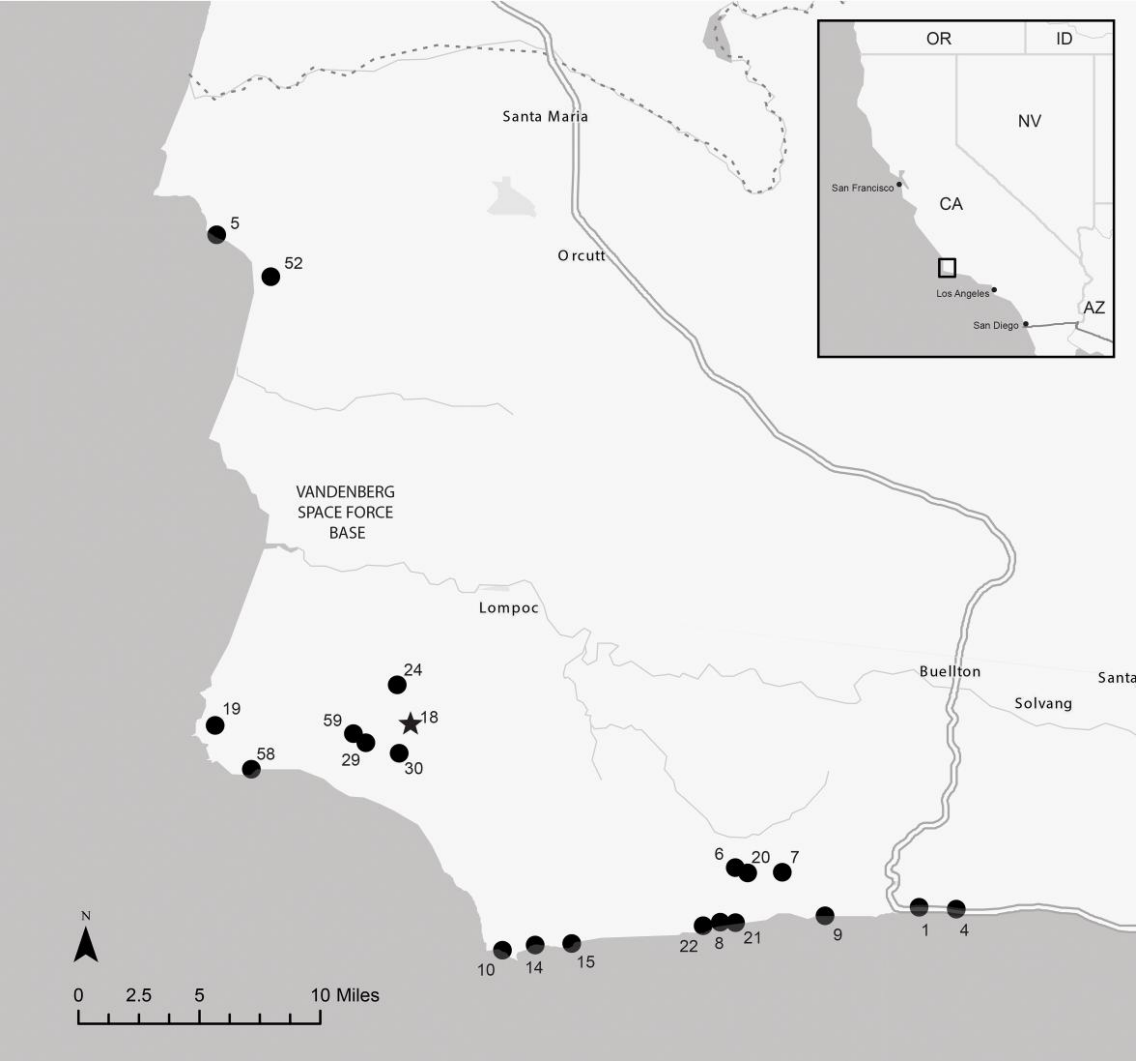
Map of southwestern Santa Barbara County, California, showing *D. increscens* subsp. *villosa* EOs as filled circles, labeled with EO number. The star symbol indicates the EO where the reference individual was collected.

*Deinandra increscens* (H.M. Hall ex D.D. Keck) B.G. Baldwin subsp. *villosa* (Tanowitz) B.G. Baldwin (Madiinae, Asteraceae), commonly referred to as Gaviota tarplant, is an annual herb (Fig. 1) endemic to a narrow band along the coast in Central California, United States (Fig. 2). With only 25 total element occurrences (EOs), the taxon was listed as Endangered under the California State Endangered Species Act (CESA; California Fish and Game Code sections 2050–2089.25) in 1990 and the United States Federal Endangered Species Act in 2000 (ESA; U.S. Code, Title 16, sections 1531–1544). According to the California Native Plant Society (California Native Plant Society 2023), the top threats to the taxon include development (11 EOs, or 44%), nonnative plant impacts (6 EOs, or 24%), and road/trail construction and maintenance (5 EOs, or 20%).

Molecular genetic approaches have long been applied to study members of subtribe Madiinae (including *Deinandra*), but to date, published studies have not included genome-scale data. The systematics and evolution of subtribe Madiinae have been extensively studied using phylogenetic analyses of Sanger sequence data sets (e.g. Baldwin 1992, 1993, 2005, 2007; Baldwin and Wessa 2000) or less commonly using microsatellites (e.g. McGlaughlin and Friar 2011). Data sets using high-throughput sequencing are in development, however. Baldwin *et al*. (2021) report the development of a data set of hundreds of nuclear loci using a high-throughput, targeted sequencing approach to examine adaptive radiation of the tribe Madieae, inclusive of subtribe Madiinae. Previous cytogenic chromosome counts including 1 sample of *D. increscens* subsp. *villosa* and multiple samples of subsp. *increscens* found diploidy with 1N = 12 (Tanowitz 1982). A large population genomics project by a subgroup of the authors is underway, using a genome resequencing approach to assist in conserving *D. increscens* subsp. *villosa* (McEvoy *et al*. 2023). Here, we present the first step in that project, the development of a high-quality reference genome for *D. increscens* subsp. *villosa*.

## Materials and methods

### Sample collection, preparation, and sequencing

Fresh leaf and stem tissue were removed in the field from 1 mature individual of *D. increscens* subsp. *villosa* from the Tranquillon Mountain/Sudden Peak occurrence, south of Lompoc, California, United States (34.575360, −120.520118, 416 m). It was vouchered on 2021 May 7 by Hasenstab-Lehman and Hazelquist (3314; Santa Barbara Botanic Garden [SBBG]). Tissue was sampled into aluminum foil and immediately placed in liquid nitrogen charged dry shipper for transportation to the SBBG, where it was stored at −80°F. The leaf tissue from this sample was used to generate all DNA sequencing data. Fresh leaf and petal tissue was collected from the same location and stored overnight on ice for flow cytometry.

#### Pacific Biosciences HiFi

High molecular weight (HMW) genomic DNA (gDNA) was extracted from a whole plant (1,600 mg) using the method described in Inglis *et al*. (2018) with modifications. Sodium metabisulfite (1% w/v) was used instead of 2-mercaptoethanol (1% v/v) in the sorbitol wash buffer and the CTAB buffer. Frozen tissue was ground in liquid nitrogen with mortar and pestle for 15 min, transferred to a 50-ml tube, and immediately mixed with 10 ml of sorbitol wash buffer. The suspension was centrifuged at 5,000×g for 5 min at room temperature, and the supernatant was discarded. Using a paintbrush, the ground tissue pellet was gently resuspended in 10 ml of sorbitol wash buffer and this wash step was repeated 7 times to remove potential contaminants that may coprecipitate with DNA. The CTAB lysis step was performed at 45°C instead of 65°C for 1 h with gentle inversion every 15 min. The final DNA yield (17.3 µg) was quantified using a Quantus Fluorometer (QuantiFluor ONE dsDNA Dye assay, Promega, Madison, WI). The size distribution of the HMW DNA was estimated using the Femto Pulse System (Agilent, Santa Clara, CA), and 55% of the DNA fragments were found to be 30 kb or longer.

The PacBio HiFi data generation was performed at UC Davis DNA Technologies Core (Davis, CA). The HiFi SMRTbell library was constructed using the SMRTbell Express Template Prep Kit v3.0 (Pacific Biosciences, Menlo Park, CA; cat. #102-182-700) according to the manufacturer’s instructions. HMW gDNA was sheared to 15–18 kb using Diagenode's Megaruptor 3 system (Diagenode, Belgium; cat. B06010003). The sheared gDNA was concentrated using 1× of SMRTbell cleanup beads for the repair and a-tailing incubation at 37°C for 30 min and 65°C for 5 min. This was followed by ligation of overhang adapters at 20°C for 30 min, a second cleanup using 1× beads, and nuclease treatment at 37°C for 15 min. The SMRTbell library was size selected using 3.1× of 35% v/v diluted AMPure PB beads (Pacific Biosciences, Menlo Park, CA; cat. #100-265-900) to progressively remove SMRTbell templates <5 kb. The resulting library was sequenced using three 8M SMRT cells (Pacific Biosciences, Menlo Park, CA; cat. #101-389-001), Sequel II sequencing chemistry 2.0, and 30-h movies on a PacBio Sequel II sequencer.

#### Oxford Nanopore Technologies

HMW gDNA was extracted from the same reference individual as described above for HiFi. For Oxford Nanopore Technologies (ONT), 700 mg of frozen tissue was used, the suspension was centrifuged at 3,500×g for 5 min, and the wash step was repeated 3 times. The extracted HMW gDNA was purified using high-salt-phenol-chloroform (PacBio) to remove coprecipitating contaminants. Purity of the gDNA was checked using NanoDrop ND-1000 spectrophotometer, and a 260/280 ratio of 1.89 and 260/230 ratio of 1.90 were observed. Total DNA yield was 2.5 µg as quantified by Qubit 2.0 Fluorometer (Thermo Fisher Scientific, MA). The integrity of the gDNA was verified on a Femto Pulse System (Agilent Technologies, Santa Clara, CA) where an average fragment size of 11 kb was observed.

The ONT sequencing library was prepared from 1.5 µg of HMW gDNA using the ligation sequencing kit SQK-LSK114 (ONT, Oxford, UK) following the manufacturer's instructions with the exception of extended incubation times for DNA damage repair, end repair, ligation, and bead elutions. A total of 30 fmol of the final library was loaded on a PromethION R10.4.1 flow cell (ONT, Oxford, UK), and the run was set up on a PromethION P24 device using MinKNOW 22.10.7. Data were collected for 72 h, and basecalling was performed during the run using ONT's basecaller ont-guppy-for-promethion 6.3.9 with the corresponding super accuracy basecalling model.

#### Dovetail Omni-C

Tissue from the reference sample was prepared using the Dovetail Omni-C Kit (Dovetail Genomics, Scotts Valley, CA). The manufacturer's protocol was followed with slight modifications. Tissue was ground in liquid nitrogen with a mortar and pestle. Nuclear isolation followed published methods (Workman *et al.* 2018). Chromatin was fixed in the nucleus and digested under various conditions of DNase I until a suitable fragment length distribution of DNA molecules was observed. Chromatin ends were repaired, ligated to a biotinylated bridge adapter, and followed by proximity ligation. Crosslinks were reversed, and the DNA was purified from proteins and treated to remove biotin external to ligated fragments. The library was generated using an NEB Ultra II DNA Library Prep kit (NEB, Ipswich, MA) with an Illumina-compatible y-adaptor. Fragments containing biotin were then captured using streptavidin beads, and the postcapture product was split into 2 dual-unique indexed replicates before PCR enrichment to preserve library complexity. The library was sequenced at the UCLA Technology Center for Genomics and Bioinformatics (TCGB; Los Angeles, CA) on an Illumina NovaSeq SP at 2X150, aiming to generate 400 million reads.

#### RNA-seq

Samples were collected from 3 individuals in the Tranquillon Mountain/Sudden Peak occurrence south of Lompoc, California, United States (GVTP_1, GVTP_2 at 34.580914, −120.514450 and GVTP_5, 34.573121, −120.522042). Shoot and root tissue samples were collected from seedlings in rosette form and immersed in liquid nitrogen followed by −80°C storage. In addition, leaf and stem tissues were collected from the adult reference individual already in freezer storage bringing the total number of samples to 8. Processing and sequencing were conducted by the UCLA TCGB. RNA was isolated from the frozen plant tissues using the QIAGEN RNeasy Plant Mini Kit (ref. #74903) using lysis buffer RLT and 2 M dithiothreitol (DTT). Library preparation of each sample was conducted using the KAPA Stranded mRNA-Seq Kit (96rxn; cat. # KK8421). Sequencing of individual samples was generated at TCGB on an Illumina NovaSeq SP at 2X150 with 2 libraries per lane, aiming for 20 M reads per library.

### Genome assembly

Genome size estimation was conducted using both cytometric and sequencing methods. Flow cytometry trials were conducted comparing leaf and petal tissue of *D. increscens* subsp. *villosa* (Ohalo Genetics; Aptos, CA, USA). Petals produced a better peak and were run using *Helianthus annuus* (sunflower) petal tissue and *Zea mays* (corn) and *Lactuca sativa* (lettuce) leaves as reference points. Resulting genome size estimates were used to predict the amounts of sequence data necessary for genome assembly. HiFi and, later, ONT data were used for sequence-based genome size estimation and ploidy using GenomeScope2 and Smudgeplot v0.2.2 (Ranallo-Benavidez *et al*. 2020). *k*-mer histograms of various sizes were created with KMC 3 (Kokot *et al*. 2017) to compare results.

Contig assembly tests were initially conducted with HiFi using the assemblers Hifiasm 0.16.1-r375 (Cheng *et al*. 2021), Flye v2.9.1-b1780 (Kolmogorov *et al*. 2019), and wtdbg2 v2.5 (Ruan and Li 2020). To improve results, ONT sequencing was generated and run in Flye (--nano-hq) and Canu v2.2 (Koren *et al*. 2017). ONT was also run in combination with HiFi reads in Verkko v1.4.1 (Rautiainen *et al*. 2023). ONT-based Flye, Canu, and ONT/HiFi Verkko assemblies were run through Purge Haplotigs v1.1.2 (Roach *et al*. 2018) using Minimap2 v2.24-r1122 (Li 2018) alignments to separate out undercollapsed haplotype duplication into primary and alternate assemblies. Of these, the Flye and Canu resulted in the best contig assemblies and were tested in the scaffolding step.

Scaffolding followed recommended methods and tools for the 3D-DNA pipeline (https://aidenlab.org/assembly/manual_180322.pdf). Omni-C reads were aligned to the contig assembly with BWA (Li 2013), and the alignment was used as input for Juicer v1.5 (Durand *et al*. 2016) to create a formatted file of duplicate-filtered long-distance pairs. This pairs file was provided along with the genome to 3D-DNA v180922 for scaffolding (Dudchenko *et al*. 2017). Tuning of parameters was evaluated by visualization of Omni-C alignment heatmaps and coverage plots in Juicebox (Durand *et al*. 2016), and “--editor-repeat-coverage 5” was selected as it produced the longest scaffolds with the least unanchored fragments. There was insufficient Omni-C data coverage over portions of the Canu contig assembly, which was the rationale for eliminating this version and proceeding with the Flye-based assembly.

The scaffolded ONT-based Flye assembly was screened for assembled chloroplast using alignment of chloroplast contigs assembled by GetOrganelle v1.7.7.0 (Jin *et al*. 2020) and the closest relative with a chloroplast reference genome, Asteraceae *Achyrachaeana mollis* (NCBI RefSeq accession NC_036504.1). Contaminant screening was done with both Blobtools2 v4.1.3 and the NCBI Foreign Contamination Screening tool FCS-GX v0.4.0 (Astashyn *et al.* 2024 ). Gap filling was done using TGS-GapCloser v1.2.1 and the ONT error-corrected reads produced by Canu during the contig assembly tests (Xu *et al*. 2020).

Throughout the workflow, contig and scaffold assemblies were evaluated with QUAST v5.2.0 (Gurevich *et al*. 2013) and BUSCO v5.4.3 (-geno) using the embryophyta_odb10 database (Manni *et al*. 2021). Final assembly completeness was also evaluated with Merqury v1.3 (Rhie *et al*. 2020). This involved removing the bridge adapters from the Omni-C reads using Cutadapt v2.6 (Martin 2011) to prepare histograms of *k*-mer counts in Meryl v.1.3 (Rhie *et al*. 2020). A full list of software and versions used in the genome assembly process is also available in Supplementary Table 1.

### Repeat annotations

A consensus of transposable elements (TEs) and other repetitive content was identified de novo throughout the final genome using RepeatModeler v2.0.4 with the -LTRStruct parameter for LTR discovery (Flynn *et al*. 2020). The resulting repeat library was combined with curated plant long terminal repeats (LTRs) in InpactorDB non-redundant v5 (Orozco-Arias *et al*. 2021) and softmasked with RepeatMasker v4.1.5 using -gff -a -xsmall (Smit *et al*. 2013). The RepeatMasker .out format file was used as input for the script ParseRM () using -p -n -l 50,1 to generate summary statistics according to TE category and to format data for the TE landscape abundance, which was plotted in ggplot2 v3.4.2 in R v4.2.1 (Wickham 2016).

### Gene annotations

RNA-seq alignments were used to improve gene prediction. RNA-seq reads were checked for quality control with FastQC v0.11.9 (Andrews 2010) and then trimmed with fastp v0.22.0 (Chen *et al*. 2018). Initial alignments with Hisat2 v2.2.1 (Kim *et al*. 2019) were lower than the desirable threshold of 80%, so the unmapped reads were screened for contaminants with Kraken2 v2.1.2 (Wood *et al*. 2019) using the MiniKraken v2 database of bacteria, archaea, and virus. Matches were removed from the subset of reads that did not map using seqtk v1.3-r106 (Kapusta 2017), and the remaining reads were combined back with the mapped readset. These improved read alignments were provided as input along with the softmasked genome for the EASEL v1.3 annotation pipeline (Webster *et al*. 2023) using the default recommended configuration for plants with the exception of a reduced “rate” parameter of 70 to include all aligned libraries. EASEL assembles reference-based transcripts using Stringtie2 (Shumate *et al*. 2022) and PsiCLASS (Song *et al*. 2019), identifies open reading frames with TransDecoder (Haas 2023) refined by EggNOG-mapper (Cantalapiedra *et al*. 2021), and uses resulting transcript and protein alignments combined with OrthoDB protein alignments for training and gene prediction with AUGUSTUS (Stanke *et al*. 2006), resulting in an unfiltered structural annotation with alternative transcripts. A matrix of primary and secondary sequence features for resulting transcripts is scored and filtered using a random forest algorithm with a plant-based training set. Final metrics are generated using AGAT (Dainat 2024), gene space completeness with BUSCO (-prot), and functional annotation with EnTAP v0.10.8 (-tcov 70, -qcov 70) using databases RefSeq complete v208 and EggNOG v4.1 (Powell *et al*. 2014; O’Leary *et al.* 2016; Hart *et al*. 2020).

### Gene duplication

To examine the gene duplication reflected in the BUSCO score of the final assembly, genome-wide gene duplication was identified and categorized using DupGen-finder with default parameters (Qiao *et al*. 2019). *Nelumbo nucifera* was used as an outgroup, and DIAMOND blastp v2.1.8 was used to generate input alignments (--sensitive --max-target-seqs 5 --evalue 1e-10; Buchfink *et al*. 2015). Genes categorized as whole genome duplication (WGD) were used to calculate and plot the frequency of duplication by synonymous mutation (Ks) to identify potential WGD events. WGD paralogs were also visualized in Circos (Krzywinski *et al*. 2009) to examine their genome-wide distribution.

## Results and discussion

### Sequencing and genome assembly

Three HiFi sequencing runs resulted in a total of 6.3 M reads with an N50 of ∼13 kb, consisting of 76.8 B bases for 46× in coverage using the assembly length and ONT-based genome size estimates. Adaptor filtering only removed 1 read. ONT sequencing resulted in 14.8 M reads and 101.2 B bases. At 61×, the coverage was a little better than HiFi, but the N50 was shorter at only 10.7 kb. Two Omni-C lanes generated a combined 848 M 150 bp PE reads with 126.8 B bases and 75.9× coverage (Supplementary File 1).

**Fig. 3.**
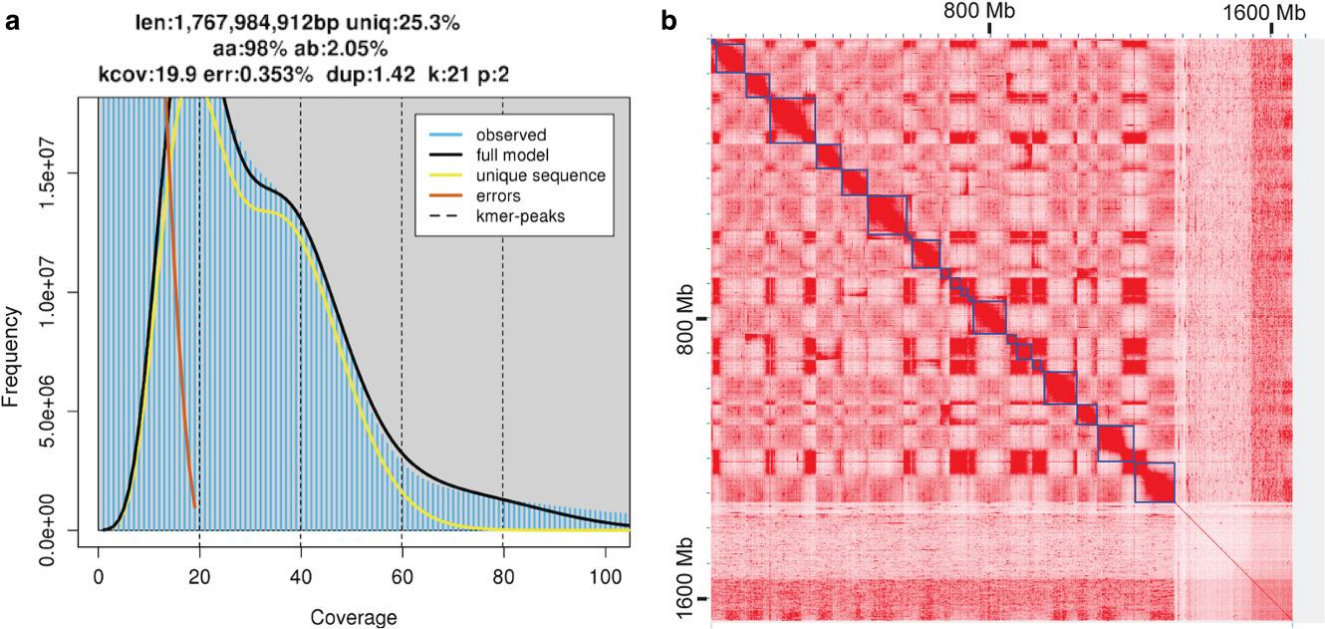
a) Genome estimates generated with HiFi sequencing using the diploid setting with *k*-mer size 21. Note the double peaks with the top of the heterozygous (first) peak cut off and overlapped by the error model (red line). Genome size was estimated at 1.77 Gb with 75% repeat content and 2% heterozygosity. b) Heatmap of Omni-C read pairs mapped to the scaffolded genome. The *X*-axis shows a total length of ∼1,650 Mb. Blue lines delineate the 21 largest scaffolds estimated by 3D-DNA. Discarded fragments and small contigs to the right show a thin line of alignment indicating an absence of significant Omni-C support for anchoring in superscaffolds.

Flow cytometry using related species of *L. sativa*, *H. annuus*, and *Z. mays* as general reference points and petal tissue of *D. increscens* subsp. *villosa* helped place it at an average 1C of 2.2 ± 0.1 pg, which translates to a base count of approximately 1.7–2.3 Gb (Supplementary File 2). This estimate was supported by sequence-based *k*-mer profiles where genome lengths in ONT trials using *k*-mers 21 and 35 were 1.61 and 1.69 Gb, and HiFi trial with *k*-mers 21, 27, and 31 ranged from 1.77, 1.76 to 1.74 Gb. As the plot in Fig. 3a demonstrates, the data were able to converge on GenomeScope models, but they were not a good fit. The heterozygous (left) peak had substantial overlap with the error model, and the very top of the peak is obscured. Further examination of the reads to determine the cause did not find evidence of excessive contamination. Polyploidy is not likely as previous limited cytogenic chromosome counts found diploidy (Tanowitz 1982), and Smudgeplot created with the same histograms all supported a diploid genome (Supplementary Fig. 1). It is possible the plots are due to inadequate read coverage and a high level of heterozygosity (2%) and repetitive content (75%) reported by the estimate and later validated by repeat annotation of the resulting assembly.

Issues with the read *k*-mer estimates were predictive of complications with contig assembly. Attempts with HiFi were not able to complete in Hifiasm and stalled during its initial *k*-mer profiling step. ONT reads were generated in the hopes that additional coverage and longer read lengths could help resolve assembly across the estimated repeat content. Though we were able to achieve greater coverage (60×), unfortunately, the ONT read N50 (10.7 kb) was even shorter than the HiFi and resulted in a fairly fragmented contig assembly in all assemblers tested. The ONT contig assembly created with Flye was 2.46 Gb in length with 101 K contigs and an N50 of 76.6 kb. Though fragmented, it was complete, with a BUSCO score of 99.2%, 79.2% of which was duplicates. Purging undercollapsed haplotypes reduced the BUSCO score to 98.3% with 22.2% duplicates. The purged primary haplotype assembly was 1.67 Gb in 37 K contigs with an N50 of 108 kb. The combined primary and alternate haplotype assemblies resulted in 71.5% completeness in Merqury with a quality value (QV) of 28.79. The alternate assembly was only 745 Mb and 75% complete in BUSCO, so it was not feasible to obtain both haplotype assemblies using Flye.

Canu with ONT likely assembled much of both haplotypes as the length was approximately twice the expected at 4.18 Gb and BUSCO found 98.4% duplicates. Unfortunately, the haplotypes did not separate cleanly using available tools, leaving a total length of 2.56 Gb and 27.9% duplicate BUSCO genes. The Omni-C reads did not provide consistent coverage across regions of this assembly resulting gaps of sufficient data and a low-quality scaffold genome.

Proceeding with the scaffolded Flye assembly, after adjustments to remove contaminants, plastid contigs, and fill gaps, the primary assembly was 1.67 Gb as 28.7 K scaffolds with a scaffold N50 of 74.9 Mb, L50 of 9, and 600 Ns per 100 kb (Table 1). Statistics for assembly iterations are available in Supplementary File 1. BUSCO completeness for the final assembly (genome mode) was 98.1% with 15.7% duplicate copies, 0.9% fragmented, and 1.0% missing. Assessment of the primary haplotype without the alternate in Merqury was 62.25% with a QV of 29.49.

**Table 1.** Genome assembly statistics.

Length	1,674,664,524
No. of contigs	28,693
Contig N50	74,894,508
Scaffold N50	76,599
Scaffold L50	9
Largest contig	132,903,021
GC content	37.27%
No. of N's per 100 kb	600.43
BUSCO embryophyta_odb10	C:98.1%[S:82.4%,D:15.7%],F:0.9%,M:1.0%,*n*:1614
Merqury—primary and alternate haplotype contig assembly	QV: 28.79, completeness: 71.48%
Merqury—primary scaffold assembly	QV: 29.49, completeness: 62.25%

Remaining fragmentation in this assembly highlights the importance of adequate long-read sequencing, particularly for plant genomes with extensive TEs. For future versions, the addition of ultralong ONT would be beneficial. Not enough sample remains for additional sequencing, but perhaps new Omni-C data from a different individual could provide enough consistent coverage to reduce fragmentation and possibly resolve the putative haplotypes observed in Canu. Examination of synteny with sunflower or linkage maps from the population-level resequencing in development could help further anchor existing scaffolds, at least those that are not primarily repeats.

### Repeat annotations

**Fig. 4.**
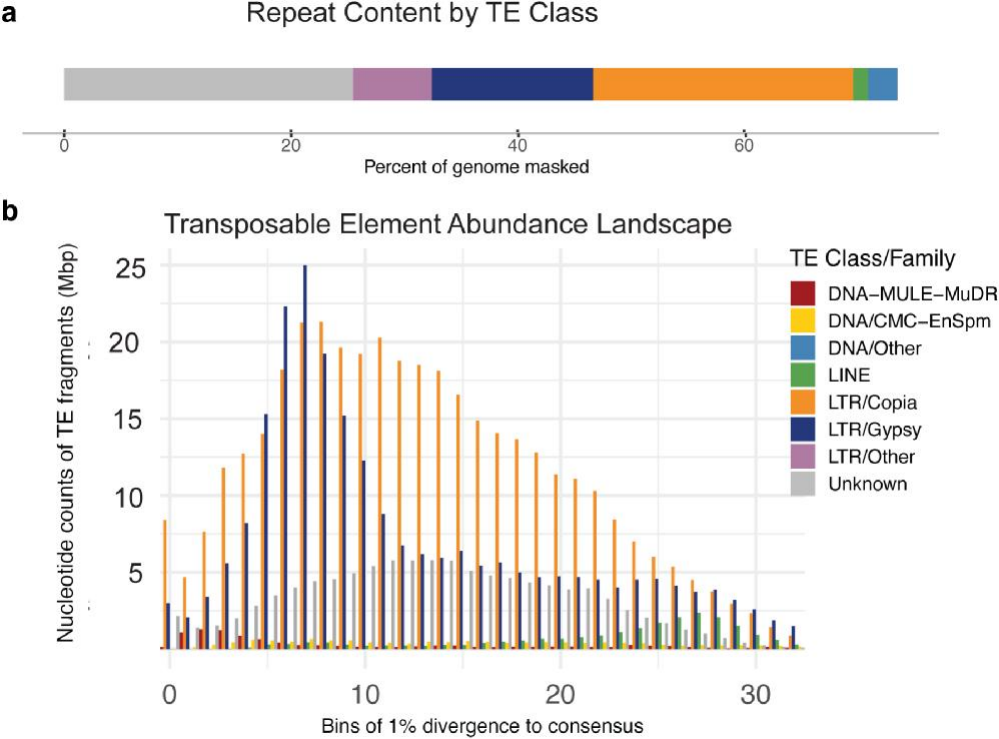
a) Percentage of base pairs masked as TEs across the *D. increscens* subsp. *villosa* genome. TE classes shown: Unknown, LTR/*Gypsy* LTR/*Copia*, LINE, and DNA, 74.8% in total. b) Abundance of TE elements as base pair totals binned by divergence as a proxy for relative time. Only the most abundant TE classes and families are plotted in this figure; the full summary of elements is found in Supplementary File 1.

Repeats were identified in all but 53 scaffolds and softmasked in 74.81% of the genome (Table 2; Supplementary File 1). LTRs covered 43.96% of the genome with *Copia* families predominant at 22.88% followed by *Gypsy* at 14.15% (Fig. 4a). There were more copies of *Copia* overall, but fewer unique, with 451,119 copies identified within 29,511 unique elements. *Gypsy* had 272,599 copies in total with 32,255 unique elements. DNA transposons masked 2.6%, led by MULE-MuDr (0.76%), CMC-EnSpm (0.59%), and PiF-Harbinger (0.56%). A TE landscape abundance plot using divergence to represent relative time of insertion revealed more ancestral activity compared to recent (Fig. 4b).

**Table 2.** Repeat annotation statistics for all TE classes and most abundant families.

Class/family	% masked
Total	73.78
DNA	2.60
DNA/MULE-MuDR	0.76
DNA/CMC-EnSpm	0.59
DNA/*PIF-Harbinger*	0.56
DNA/*Zisupton*	0.21
LINE	1.31
LTR	43.97
LTR/*Copia*	22.88
LTR/*Gypsy*	14.15
PLE	0.01
RC	0.34
SINE	0.12
Unknown	25.43

Interestingly, elements found most frequently within these ancestral peaks of abundance were from a mix of classes and families including both LTRs *Gypsy/Tekay* and *Copia/Sire*. Two of the most prevalent elements in these peaks were unclassified with only small sections of sequence similarity to anything within ASTER-REP, a curated Asteraceae-specific TE database created from genome skimming (Ventimiglia, Castellacci, *et al.* 2023; Ventimiglia, Marturano, *et al.* 2023). Recently active TEs were also a mix led by *Copia/Angela*, *Gypsy/Tekay*, and unclassified elements. *Copia/Angela* elements were more accurately identified by sequence similarity using *Helianthus* elements in the ASTER-REP database (Ventimiglia *et al*. 2022). The element with the greatest peak of abundance was originally identified as *Gypsy* by RepeatMasker but was also found by sequence similarity to have an internal *Copia/Angela* element. Such complex, nested, or unknown elements are likely indicative of the long history and high level of repeat content found within the lineage.

The amount and composition of TE content in a genome are due to the success of different TEs active within that genome. While genomic studies do not exist for most Asteraceae, comparisons of TEs exist for important crop species including *L. sativa*, *Cynara cardunculus* var. *scolymus* (artichoke), *Artemisia annua* (sweet wormwood), *Carthamus tinctorius* (safflower), and *Chrysanthemum seticuspe* (Ventimiglia, Castellacci, *et al.* 2023; Ventimiglia, Marturano, *et al.* 2023). In particular, extensive work exists on *H. annuus* (Badouin *et al*. 2017; Mascagni *et al*. 2017; Ventimiglia, Castellacci, *et al.* 2023; Ventimiglia, Marturano, *et al.* 2023), which is more closely related and has a divergence time of 26.9 MYA from *D. increscens* (Kumar *et al*. 2017). *Helianthus annuus* and *D. increscens* subsp. *villosa* are both at the high end of total repeat content for Asteraceae, at almost 80%. They are also similar in LTR proportion (∼42%), which falls midrange for these Asteraceae (35.27% in *C. cardunculus* var. *scolymus* and 52% in *C. seticuspe*). But ratios of *Gypsy* to *Copia* vary dramatically across Asteraceae with some favoring 1 family over the other. *Helianthus annuus* is on one end of the Asteraceae spectrum, with a ratio of 6.47 *Gypsy:Copia* (only 6.35% *Copia*), and *D. increscens* subsp. *villosa* is on the other with 0.62, which further supports the hypothesis that different LTR sublineages had different rates of activity after speciation (Ventimiglia, Castellacci, *et al.* 2023; Ventimiglia, Marturano, *et al.* 2023). TE activity has greatly influenced genome evolution in both these species with very different results.

### Gene annotations

After contaminant filtering, read counts of the 8 RNA-seq libraries ranged from 81 to 130 M with alignment rates ranging from 76.0 to 91.42%. From this, StringTie generated 76,471 transcripts while PsiCLASS produced 116,777. These transcripts were used as nucleotide and protein evidence for training AUGUSTUS in preparation for gene prediction. After prediction, unfiltered transcripts identified by the EASEL pipeline totaled 334 K with 197 K genes and contained many false positives as indicated by the high mono- to multiexonic rate (0.69; Vuruputoor *et al*. 2023) and the low frequency of genes identified by sequence similarity rate in EnTAP (0.65). False positives were well resolved by EASEL's extensive filtering algorithms, which produced 68,942 final transcripts and 33,257 genes. The final gene set had a more typical monoexonic/multiexonic rate of 0.27, and EnTAP results were greatly improved, identifying 92% by sequence similarity (Table 3; Supplementary File 1). Over 99% of the transcripts had functional annotations provided by sequence similarity and/or gene family assignment. BUSCO run in protein mode resulted in 96.0% complete genes with an 18.4% duplication rate, slightly higher than that of the assembly (15.7%). Despite the large number of assembly scaffolds, genes are only present in 2,444 scaffolds and 91% of the total genes are consolidated in the 22 largest. Given the previous cytogenic chromosome counts of 1N = 12 (Tanowitz 1982), these 22 contigs may represent significant portions of pseudochromosomes. The remaining 26 K scaffolds without genes likely contain repeats that need to be bridged by ultralong reads for greater contiguity.

**Table 3.** Gene annotation statistics.

Filtered: total number of genes	33,257
Total transcripts	68,942
EnTAP sequence similarity rate	0.92
EnTAP gene family assignment rate	0.99
Monoexonic/multiexonic rate	0.27
BUSCO (embryophyta_odb10)	C:96.0%[S:77.6%,D:18.4%],F:0.6%,M:3.4%,*n*:1614

### Gene duplication

**Fig. 5.**
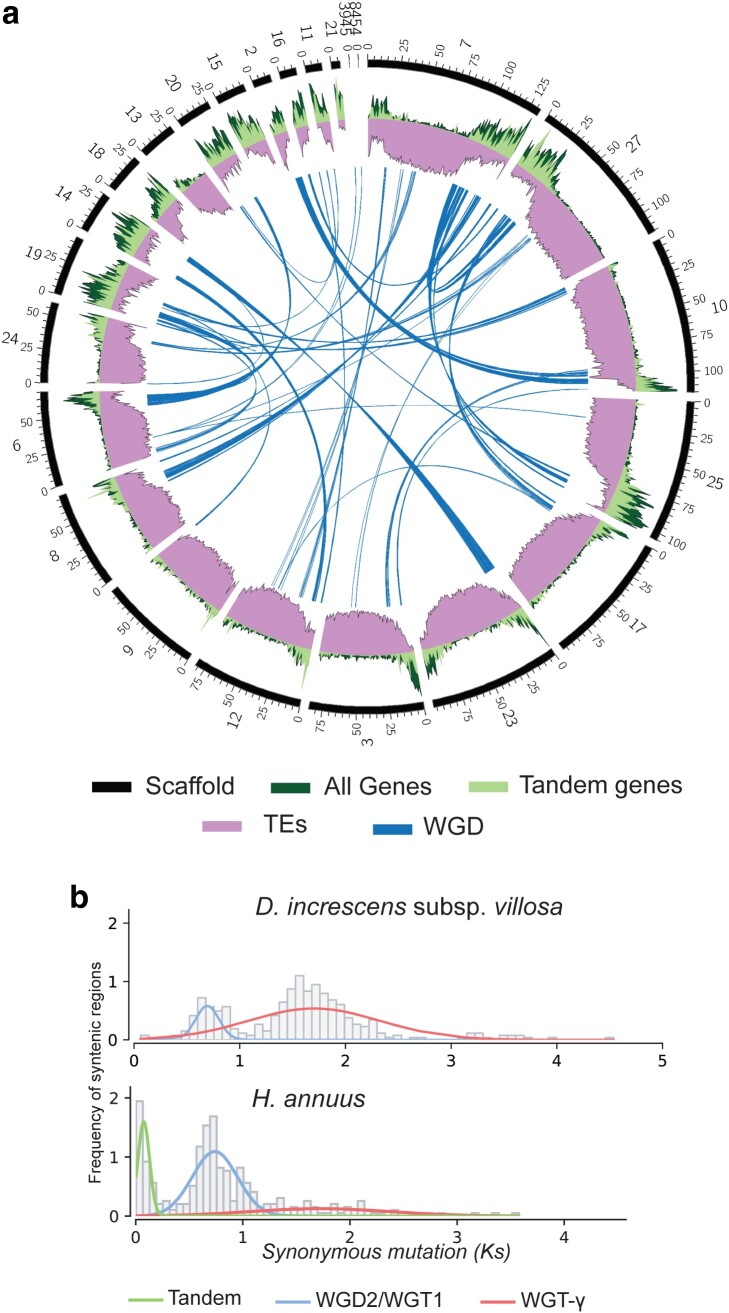
a) Circular plot representing the 22 largest scaffolds (black) arranged clockwise by size, plus 2 others, all containing WGD. Syntenic blocks of gene pairs are linked in blue. Gene density (counts per 100 Mb window) is plotted in dark green and overlaid by the density of tandem genes in light green. TE frequency as a percentage of base pairs per window is shown in purple. b) Peaks representing WGD events in *D. increscens* subsp. *villosa* (top) and *H. annuus* (bottom). Peaks consist of an increased frequency of syntenic blocks, which are regions of collinear genes in proximity, by the rate of observed average rate of synonymous mutations for the blocks.

Protein-coding gene pairs were grouped into the following categories: 5,261 WGD pairs (8,270 genes), 2,515 tandem pairs (3,583 genes), 2,312 proximal pairs (3,526 genes), 6,375 transposed pairs (5,982 genes), and 23,898 dispersed pairs (31,759 genes). Plotting links between the WGD gene pairs revealed that they are mostly, but not exclusively, found in gene-dense regions on the 22 largest scaffolds plus 2 others that are very short (Fig. 5a). The WGD pairs produced 2 peaks of elevated frequency at synonymous mutation rates (Ks) 0.69 and 1.70 that aligned approximately with peaks in *H. annuus* at 0.59 and 1.81 (Fig. 5b). In *H. annuus*, Badouin *et al*. (2017) used paralogs and homologs from speciation events to identify the most ancient peak as the well-established core eudicot whole genome triplication (WGT)-ɣ event. The more recent peak is 2 overlapping polyploidization events from the Asteraceae lineage: WGT-1 at 38–59 MYA and WGD-2 at 29 MYA. The most recent peak in *H. annuus* (the green line in Fig. 5b) was reported by Badouin *et al*. (2017) as tandem genes. Our method removed non-WGD genes, such as tandems, before plotting Ks values, so this peak is not portrayed for *D. increscens* subsp. *villosa*. Regions of tandem gene density are presented across the 22 largest scaffolds in Fig. 5a, where the greatest density can be observed primarily toward the ends of the 12 largest scaffolds and across the full length of the smaller scaffolds that are likely missing similar but unassembled regions of high repeat frequency with low gene density (Fig. 5a).

WGD and tandem genes differ in their evolutionary consequences. In angiosperms, WGD events have been shown to be followed by diversification of species (Landis *et al*. 2018), which could in part explain the timing of the divergence of *H. annuus* and *D. increscens* lineages after the 2 more recent whole genome events. WGD genes and tandem genes can play significant and sometimes complementary roles in adaptation to environmental changes (Guo *et al*. 2022). They may also have functional bias, with tandem genes containing lineage-specific adaptations to environmental stimuli in areas of stress response and disease resistance as compared to the more regulatory roles of WGD genes as observed in *Populus trichocarpa* and *Arabidopsis thaliana* (Hanada *et al*. 2008; Rodgers-Melnick *et al*. 2012; Qiao *et al*. 2019).

### Summary

In this study, we leveraged long-read DNA sequencing and Hi-C data to generate a scaffold-level reference genome assembly for the highly repetitive and heterozygous *D. increscens* subsp. *villosa*. The reference includes repeat annotations and protein-coding gene annotations, along with high-level characterization of extensive gene duplication throughout the genome. Larger genomes with a high percentage of repeats can result in assemblies not achieving chromosome-level status as was seen in the related *H. annuus*, which has a similar total repeat content. While the haploid version of the *D. increscens* subsp. *villosa* genome presented here is highly fragmented, much of the fragmentation seems to exist within the repeat space and the majority of gene space is consolidated within the 22 largest scaffolds. Complexity of repeat content and heterozygosity could be further resolved with ultralong read sequencing. While the total repeat content is similar to *H. annuus*, TE lineage abundances are dramatically different, indicating activity of a variety of TE classes and families throughout time with very different outcomes. Tandem and WGD are also described within the context of *H. annuus* and other Asteraceae, revealing evidence of the WGD and WGT events shared by this family and likely occurring only a few million years before divergence of the *D. increscens* subsp. *villosa* lineage. Clusters of tandem gene duplications are also interesting and could be a source of defense response and other environmental stress-related genes. Whole genome annotation of genes, repeats, and gene duplications will provide a valuable reference for future analyses and more accurate results for current efforts involving resequencing the broader EOs for genetic and genomic insights to inform conservation monitoring and mitigation.

## Supplementary Material

jkae117_Supplementary_Data

## Data Availability

Data are available under NCBI BioProject ID PRJNA1046654 with reference genome reads in BioSample SAMN38504800 and additional RNA-seq in BioSamples SAMN39983642–7. The Whole Genome Shotgun project has been deposited at DDBJ/ENA/GenBank under the accession JBCNJP000000000. The version described in this paper is version JBCNJP010000000. Methods and commands are at https://doi.org/10.5281/zenodo.10702998. Supplemental material available at G3 online.
