# Supplementary material for: The reference genome of an endangered Asteraceae, Deinandra increscens subsp. villosa, endemic to the Central Coast of California
Source: G3 (Bethesda). 2024 Jun 7;14(8):jkae117. doi: 10.1093/g3journal/jkae117 (PMC11304951; doi:10.1093/g3journal/jkae117)
Supplement: jkae117_Supplementary_Data [file jkae117_supplementary_data.zip › Table_S1_G3-2024-404921.docx]

Table S1: Assembly Pipeline and Software Used. Software citations are listed in the text.

| **Genome Contig Assembly** | | |
| --- | --- | --- |
|  | FASTQC | 0.11.8 |
|  | FASTP |  |
| Kmer counting | KMC | 3.1.1 |
|  | GenomeScope | 2 |
|  | Smudgeplot | 0.2.2 |
| *De novo* assembly | Flye | 2.9.1-b1780 |
| Long-read alignment | Minimap2 | 2.24-r1122 |
| Separate haplotype duplication | Purge Haplotigs | 1.1.2 |
| **Genome Scaffold Assembly** | | |
| Omni-C mapping | Juicer | 1.6 |
| Contig scaffolding | 3D-DNA | 180922 |
| Scaffold visualization | Juicebox tools | 1.11.08 |
| Contaminant screening | Blobtoolkit | 4.1.3 |
| Contaminant screening | Foreign Contamination Screening FCS-GX | 0.4.0 |
| Plastid screening | GetOrganelle | 1.7.7.0 |
| Gap closing | TGS-GapCloser | 1.2.1 |
| Assembly statistics | QUAST | 5.2.0 |
| Assembly completeness | Cutadapt | 2.6 |
| Assembly completeness | Meryl | 1.3 |
| Assembly completeness | Merqury | 1.3 |
|  |  |  |
| Assembly completeness | BUSCO and embryophya odb10 | 5.4.3 |
| **Repeat Annotation** | | |
| Repeat identification | Repeat Modeler | 2.0.4 |
| Repeat Masking | Repeat Masker | 4.1.5 |
| Repeat Masking | InpactorDB_non_redundant_final_V5 | 5 |
| Repeat Masker parsing | ParseRM | N/A |
| **Gene Annotation** | | |
| RNA-Seq Contaminant screening | Kraken | 2.1.2 |
| RNA-Seq filtering | Seqtk | 1.3-r106 |
| RNA-Seq alignment | Hisat2 | 2.2.1 |
| Alignment sort/merge | Samtools | 1.16.1 |
| Gene annotation | EASEL Dependency citations: https://gitlab.com/PlantGenomicsLab/easel-benchmarking-v2-nf/-/blob/main/CITATIONS.md | 1.3 |
| Functional annotation | EnTAP | 0.10.8 |
| **Whole Genome Duplication** | | |
| Identify and categorize gene duplication | Dupgene-finder/Scripts_for_GB |  |
|  | Diamond | 2.1.8 |
| Collinearity | https://github.com/reubwn/collinearity |  |
| Plot genome infor | Circos |  |
