# Supplementary figures and images for: The reference genome of an endangered Asteraceae, Deinandra increscens subsp. villosa, endemic to the Central Coast of California
Source: G3 (Bethesda). 2024 Jun 7;14(8):jkae117. doi: 10.1093/g3journal/jkae117 (PMC11304951; doi:10.1093/g3journal/jkae117)

# Smudgeplots

## HiFi K21

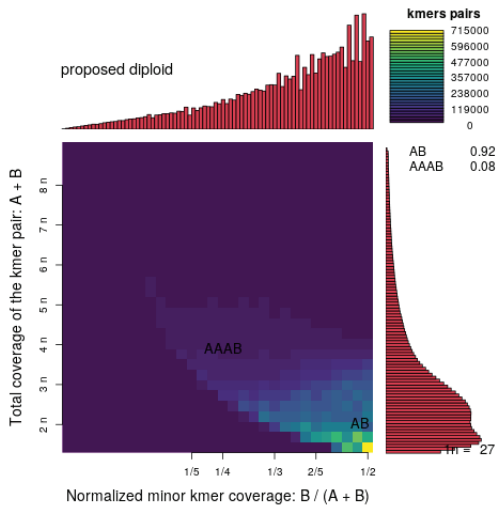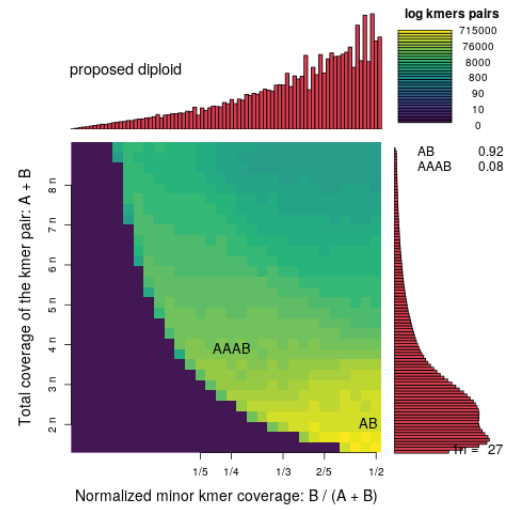

## HiFi K27

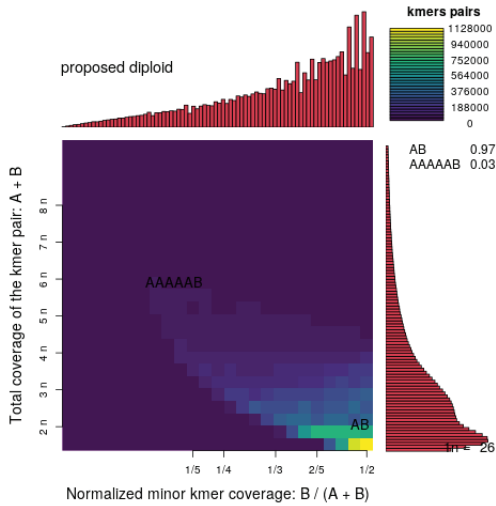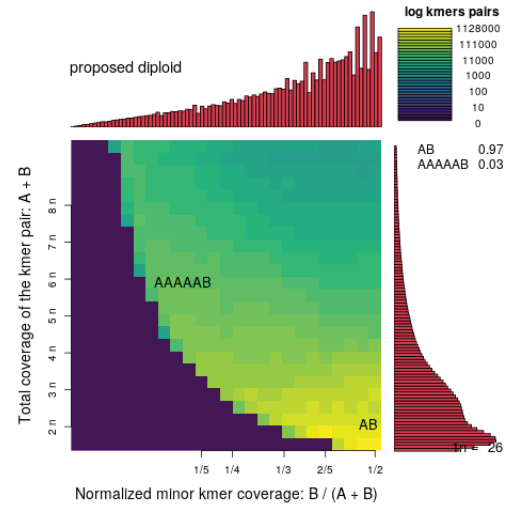

## HiFi K35

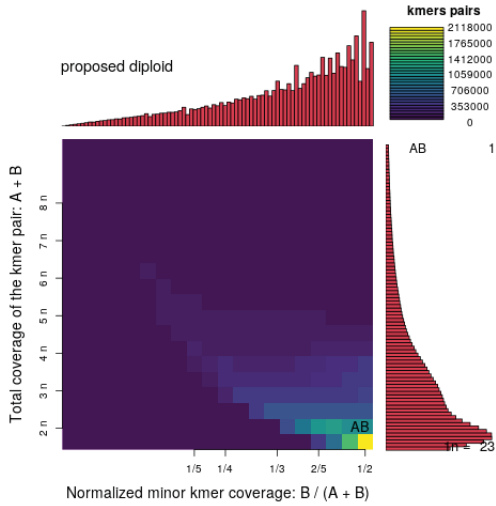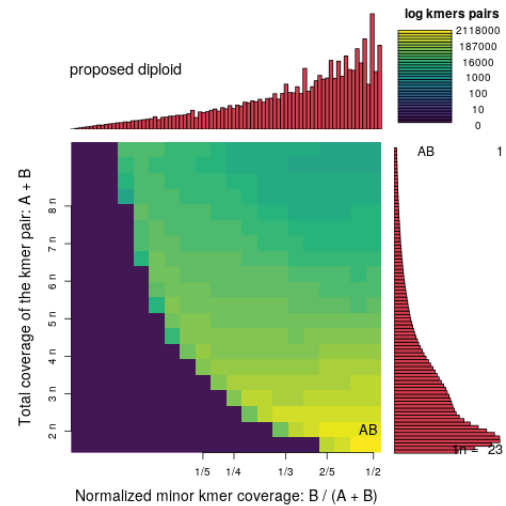

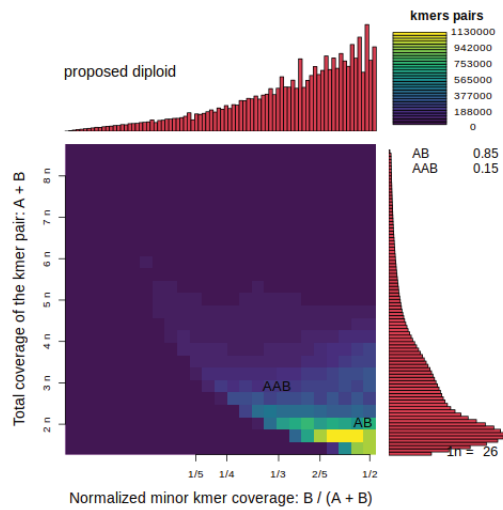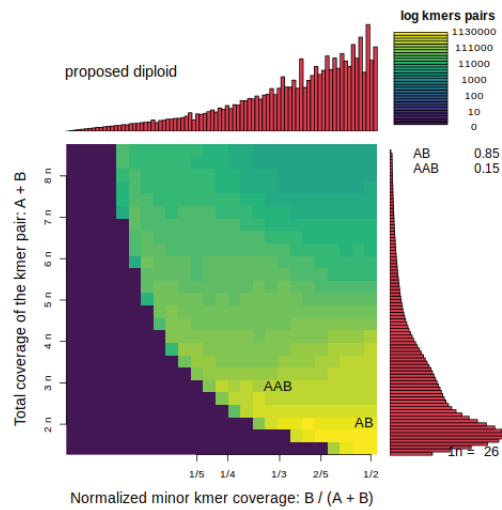

## ONT K35

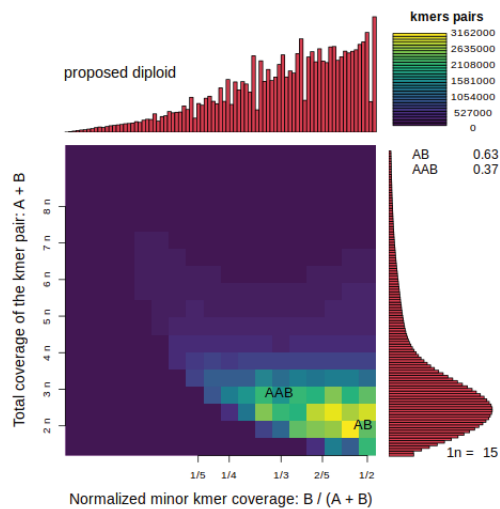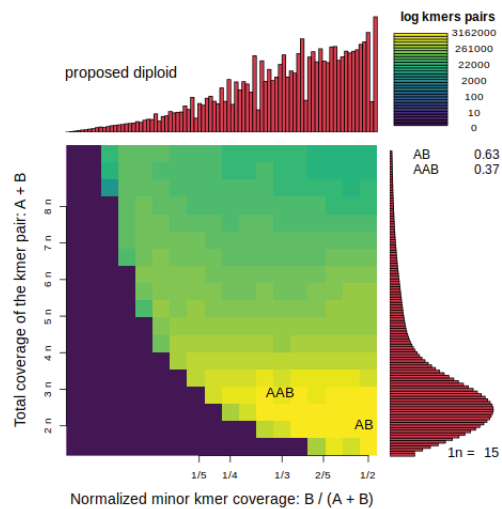

Supplement: jkae117_Supplementary_Data [file jkae117_supplementary_data.zip › Figure_S1_G3-2024-404921.pdf]
